# So‐far overlooked HFpEF patients with normal natriuretic peptide level need more evidence

**DOI:** 10.1002/ehf2.14047

**Published:** 2022-06-21

**Authors:** Atsushi Tanaka, Koichi Node

**Affiliations:** ^1^ Department of Cardiovascular Medicine Saga University Saga Japan

Community‐based cohort studies have shown that low levels of N‐terminal pro‐B‐type natriuretic peptide (NT‐proBNP) increase the risk of cardiovascular events and death.^1^, ^2^ However, these studies did not fully characterize the detailed clinical features, such as cardiac function and heart failure (HF) status, of the participants who were vulnerable to adverse prognosis due to their lower NT‐proBNP levels. Because NT‐proBNP levels are substantially affected by cardiac and non‐cardiac factors, there was a need to evaluate the prognostic value of NT‐proBNP levels in specific disease populations.

In a recent issue of the mother journal, Verbrugge *et al*.^3^ revealed the cardiac/haemodynamic characteristics of patients with HF with preserved ejection fraction (HFpEF) and normal NT‐proBNP levels, compared to patients with non‐cardiogenic dyspnoea or HFpEF with elevated NT‐proBNP. Intriguingly, NT‐proBNP level alone did not necessarily distinguish between patients with and without HFpEF, especially in the lower range of NT‐proBNP concentration, suggesting needs for alternative biomarkers more reflecting that clinical condition. The investigators also found that patients with HFpEF and normal NT‐proBNP levels also exhibited a higher risk of mortality and hospitalization for HF than that in the control subjects (patients with non‐cardiogenic dyspnoea). The study clearly raised an importance to identify the clinically meaningful characteristics of patients with HFpEF and lower NT‐proBNP and emphasized an urgent need to determine the optimal clinical management of these patients.

Recently, clinical trials of newer HF drugs, such as sacubitril valsartan and sodium‐glucose cotransporter 2 inhibitors, have pried open the tightly closed doors to effective treatments for the HFpEF population.^4^ These trials often used the inclusion criterion of elevated natriuretic peptide (NP) levels to identify true HF patients and ensure the risk of HR‐related events, and a cutoff value for HFpEF risk enrichment of 360 pg/mL NT‐proBNP or higher was suggested.^5^ Accordingly, the treatment effects observed in these clinical trials were for a *special* patient population, and it is unclear whether the findings can be applied to patient populations that were excluded, even with the same disease phenotype. The treatment effects of some drugs that have not been shown to be effective in these *special* HFpEF populations are also uncertain in *non‐special* HFpEF populations.

Importantly, we need to be reminded of the current framework in which the findings of clinical trials conducted on *special* patient populations are often used to develop treatment recommendations in relevant guidelines and consensus statements. We currently have little evidence to manage the clinically important but overlooked HFpEF population, including patients with lower NP concentrations.

In the study by Verbrugge *et al*.,^3^ the baseline condition of normal NP HFpEF, relative to non‐HFpEF, was an independent predictor of long‐term adverse outcomes (median 32 months). However, much is still unknown about the natural disease course with respect to NP levels and cardiac/haemodynamic function in that patient population. Whether this population is unique or will eventually transition to HFpEF with high NP is also unclear. Further studies are warranted to clarify the clinical manifestations and optimal clinical management for this *cannot‐miss* HFpEF phenotype.
